# Shisha Consumption and Presence of Cotinine in Saliva Samples among Students in Public Universities in Coastal Kenya

**DOI:** 10.1155/2024/5653709

**Published:** 2024-08-21

**Authors:** Abdulrehman Halima Allahdad, Moses Ngari, Cromwell Mwiti Kibiti, Rahma Udu Yusuf, Sylvia Mutua, Valentine Budambula

**Affiliations:** ^1^ Department of Environment and Health Sciences Technical University of Mombasa, Mombasa, Kenya; ^2^ KEMRI-Wellcome Trust Research Programme, Kilifi, Kenya; ^3^ Pwani University, Kilifi, Kenya; ^4^ Department of Pure and Applied Sciences Technical University of Mombasa, Mombasa, Kenya; ^5^ Department of Mass Communication Studies Technical University of Mombasa, Mombasa, Kenya

## Abstract

**Background:**

Despite the well-known adverse health effects of tobacco, shisha use among students in tertiary institutions remains a public health concern. In Kenya, the literature on status of shisha after the 2017 ban is scanty. This study sought to ascertain actual shisha use among university students along the coastal strip.

**Methods:**

We investigated confirmed and self-reported shisha use. Using proportionate-to-size and snowball sampling methods, 380 respondents were enrolled from three universities. Sociodemographic characteristics and self-reported history of shisha use were documented using a participant-assisted questionnaire. Actual shisha use was determined qualitatively using 6 panel plus alcohol saliva test kit that detected cotinine use among other selected drugs.

**Results:**

Of the 380 participants, 278 (73%) were males and their median (IQR) age was 22 (20–23) years. This study reports 29% current use based on testing positive for cotinine. Among those who reported current ever use of shisha, 19% tested positive for cotinine, respectively. In the multivariable analysis, being separated (adjusted risk ratio (aRR): 2.06 (95% CI: 1.45–2.94)) compared to being single and studying for a degree compared to a diploma (aRR: 1.32 (95% CI: 1.10–1.58)) were associated with cotinine positive. The 4^th^ year of study (aRR: 1.68 (95% CI: 1.22–2.33)) compared to the 1^st^ year and reported knowledge of shisha (aRR: 1.84 (95% CI: 1.18–2.87)) were associated with cotinine positive.

**Conclusion:**

Nearly one-third of university students along the Kenyan coast are active shisha users. Saliva testing for cotinine is a more reliable method of reporting tobacco use. We recommend upscaling of health education, re-enforcement of the current ban on shisha consumption by concerned authorities, and saliva testing for cotinine while assessing current tobacco use.

## 1. Introduction

The tobacco epidemic remains a critical public health problem as it accounts for eight million deaths per year with about 80% of current tobacco users living in sub-Saharan Africa [1, 2]. If no interventions are put in place, the number of deaths attributed to tobacco exposure is projected to double by the year 2030 in low- and middle-income countries [3].

Waterpipe tobacco smoking (WTS) also known as shisha is a rapidly emerging trend among young consumers [3, 4] especially in the Middle East and North Africa [5] partly due to the aromatic flavours in shisha [6]. Most shisha consumers believe it is less harmful compared to other forms of smoked tobacco [7]. Shisha smokers are likely to inhale more tobacco smoke than from a cigarette because of the large waterpipes used and longer exposure [8]. Tobacco smoke generated from shisha has a number of chemicals that are known carcinogens and tumour promoters [9]. The process of shisha preparation and the structure of a shisha hooker have been described in our previous work [10].

Shisha use among university students is rampant and relatively well documented. In the Kingdom of Saudi Arabia (KSA), a cross-sectional study among male university students in Buraidah city reported 46.63% overall prevalence of shisha smoking [11]. This was much higher than what was reported among university students in the Eastern Province of KSA where a 22.8% prevalence was documented [12]. At Jazan University, on the southwest coast of the KSA, the prevalence of WTS was 34.0%. The prevalence rate was significantly higher in males (42.5%) than females at 27.0% [13]. In Iran, at Tehran University of Medical Sciences, lifetime, last one year, and last one-month prevalence rates of hookah smoking were 26.6%, 17.8%, and 8.9%, respectively [10]. Based on a meta-analysis of 37 observational studies, the pooled prevalence of lifetime WPS was 25% [14].

A systematic review on WTS and cigarette smoking among university students in twelve Arab Countries [15] reported that the overall highest rate of current smoking among students was in Egypt (46.7%), Kuwait (46%), and KSA (42.3%). A study carried out among university students in three Eastern Mediterranean countries, namely, Egypt, Jordan, and Palestine, reported the highest prevalence. In this study, 60.7%, 67.7%, and 63.1% of students from Egypt, Jordan, and Palestine, respectively, self-reported having engaged in waterpipe smoking in the last thirty days [16]. In Egypt, 17.6% of medical students at Cairo University self-reported current use of shisha [17] while students at El-Sheikh University reported a lifetime prevalence of 43.6% and 22.5% current shisha use [18].

Overall, the North Africa and Middle East (MENA) region has one of the highest documented current and lifetime shisha use. Some of the key predictors of WTS among university students are being male, having a high stipend, being enrolled in a faculty of engineering, believing that shisha smoking is less harmful than cigarettes, peer influence, cigarette smoking, younger age of initiation, and having divorced parents and a mother who smokes WTS [7, 13, 18].

In sub-Saharan Africa, shisha use is scantly documented. At the University of Port Harcourt in Nigeria, a lifetime prevalence of 24.7% shisha use was reported [14]. In Burkina Faso, a study on shisha use at Université Saint Thomas d'Aquin in Ouagadougou reported a prevalence of 38.5% and incidence of 14.0% [6]. On the other hand, a cross-sectional survey among Health Science Students across 13 universities in Sudan reported 33.7% ever use of shisha [19]. In Rwanda, the prevalence among students from a private university in Kigali was 26.1% and that of those who had smoked in the last one month was 20.8% [20]. Last but not least, in Kenya, a study at the College of Health Sciences at the University of Nairobi (UoN) reported 21.5% current shisha use and a lifetime use of 34.1% [21].

In SSA, key predictors of shisha use are being male, ages between 21 and 25 years, a Muslim, in a senior year of study, and having a relatively high stipend. Additionally, peer influence, family members who use shisha, poor knowledge of health effects of shisha, and living off campus increase the risk of shisha use. Anxiety, stress, depression, use of alcohol, and other substances also predict shisha use [20–23].

Currently, research on shisha use is scanty as Kenya implemented a comprehensive ban on shisha including its use, import, manufacture, sale, offer of sale, advertising, promotion, and distribution in 2017 [24]. Despite this comprehensive ban on shisha and prohibition of drug use within premises of learning institutions [25], shisha consumption remains rampant in Mombasa as well as learning institutions. Prior to the ban, a national status on shisha and tobacco use report indicated 9.1% of Kenyans were consuming tobacco products [26].

Compared to the MENA region, shisha use among university students in SSA is less explored, hence the need to carry out this study. Moreover, the literature available for both MENA and SSA is based on self-reported history that is prone to social desirability and memory recall biases. Additionally, in Kenya, the literature on status of shisha after the 2017 ban is scanty. It is for this reason we sought to ascertain actual shisha use among university students along the coastal strip.

## 2. Materials and Methods

### 2.1. Setting, Design, and Study Participants

**Figure 1:**
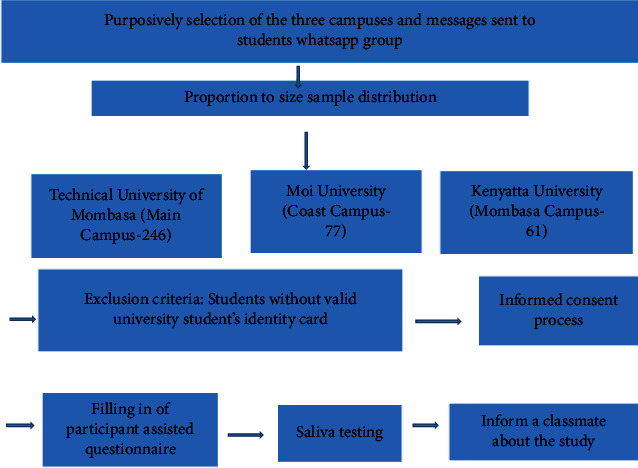
Flowchart of the study on shisha use among students in public universities in Coastal Kenya between 2018 and 2019.

This was part of a larger cross-sectional descriptive study that sought to assess the extent of substance among university students along the coastal strip of Kenya between 2018 and 2019. We enrolled students from three public universities, namely, Technical University of Mombasa (Main Campus), Moi University (Coast Campus), and Kenyatta University (Mombasa Campus), as summarized in Figure 1.

### 2.2. Ethical Considerations

The protocol for this study was ethically reviewed and approved by Pwani University Ethical Review Committee (ERC/Msc/010/2018). Participant enrollment was voluntary and through written consent. Prior to consenting, participants were educated on the objectives of the study and their right to voluntary participation as well as withdrawal from the study. Enrollment was carried out within university health clinics that provided privacy and confidentiality. The participant-assisted questionnaires were coded to avoid use of names. Both hard and soft versions were stored and have restricted access.

### 2.3. Variables and Data Source

The exposures were sociodemographics, awareness level on shisha, and use of other tobacco products. The study outcomes were reported shisha use status categorized into three levels as ever use, current use, and never use. The saliva sample tested for shisha use was reported as a binary outcome, either positive or negative.

### 2.4. Sample Size Determination and Sampling

The study enrolled 384 participants using *N*=(1.96)^2^ × 0.5(1–0.5)/0.052=384 [27]. Our attribute under study (*P*) was 0.5 since at the time of enrollment, half of the residents of Mombasa County had reported a lifetime prevalence of at least one substance [28]. At the time of the study period, the total population of TUM, KU, and Moi University was 16,000, 5000, and 3000, respectively. Therefore, proportion to size sampling based on the ratio of 16 : 5 : 4 was used to calculate the representative sampling size from each university to make the total sample size of 384 participants. The snowball sampling method was used to enroll the participants. These two sampling methods have been used elsewhere in similar subpopulation [29, 30]. Although snowball sampling method is a nonprobability sampling method, in research targeting difficult-to-reach population such as those using illegal drugs like shisha, it is considered irreplaceable and necessary sampling method.

Students were informed about the study through their WhatsApp group. This provided a fair chance of voluntary participation into the study. Each participant was verified by providing a valid university identity card. To ensure confidentiality, enrollment was carried in an enclosed consultation room. Participant-assisted questionnaire was administered by a trained research assistant. Upon successful recruitment in the study, each participant was requested to tell someone from their class. Out of the 384 participants, one had invalid results and declined to be tested again; two declined to consent to saliva testing; and one participant-assisted questionnaire was incomplete. The four participants were, therefore, excluded in the final analysis and had a total of 380 participants distributed as follows: 220 (58%); 100 (26%); and 60 (16%) from TUM, KU, and Moi universities, respectively.

### 2.5. Data Collection Tools and Procedures

A participant-assisted questionnaire was used to document social demographic characteristics, risk awareness level on shisha, and self-reported use of shisha. The tool was pretested at TUM, Kwale Campus. Confirmed drug use was carried out using 6 panel plus alcohol saliva rapid test kits as per manufacturer's instructions. The test kit is an immune-chromatographic assay that uses monoclonal antibody-coated gold particles. The test kit comprises of mouth swab sponge and a test cube. The mouth swab sponge was placed by a research assistant on the tongue or near the cheek to soak it in saliva until saturated (when the indicator strip appeared red). The saturated mouth swab sponge was placed in the test cube and closed tightly. Results were read after five minutes. The appearance of one red line in the control meant a positive result while the appearance of two lines (one at a control panel and the other at a test panel meant negative results). When no red line appeared or one red line appeared at the test panel, it meant the results were invalid and the participant was requested to consent to a repeat test. The test device detected the presence of cotinine (a metabolite of nicotine) alongside amphetamines, *Cannabis*, benzodiazepines, opiates, cocaine, and alcohol.

### 2.6. Statistical Methods

Study data were collected using a paper questionnaire and later entered into the Epidata database. We did not assume missing data were at random; for any missing data, we added an extra category (unknown/missing) and included it in the analysis.

Chi-square test of association or Fisher's exact test was used to compare the three levels of reported shisha use and different exposures. Using the saliva test for cotinine as the gold standard, we calculated the sensitivity and specificity of reporting currently using shisha. Combining those who reported currently and ever used shisha as one group (shisha use), we also calculated their sensitivity and specificity. To identify factors associated with testing positive for cotinine, we used a multilevel log-binomial regression analysis with the recruiting university as a random intercept (to account for inter-university variation). We used backward stepwise approach to select factors to include in the multivariable model. The approach starts with a full (saturated) model and only retains factors with a *P* value <0.1. As sensitivity analysis, we categorized reported shisha use into two levels: (a) no shisha use and (b) shisha use (currently using shisha plus the ever used). We similarly used multilevel log-binomial regression analysis to identify factors associated with shisha use. The regression coefficients were log-transformed and reported as risk ratios and their respective 95% confidence intervals. Statistical significance was at *α* < 0.05. Statistical analysis was conducted using STATA version 17.0 (College Station, Texas 77845, USA).

## 3. Results

**Figure 2:**
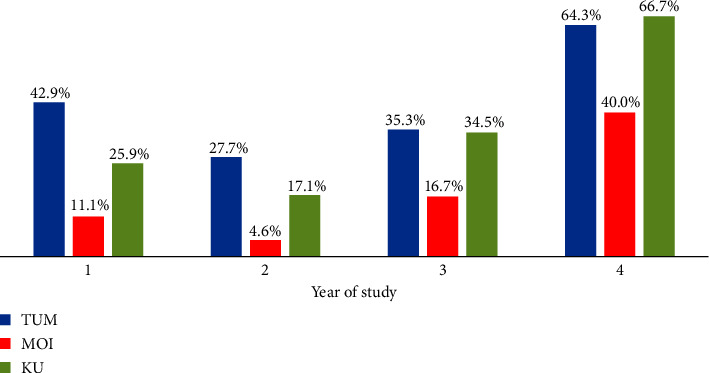
Proportion who tested positive for cotinine across the year of study stratified by the three public universities in Coastal Kenya in 2018-2019.

Of the 380 participants, 112/380 (29%) tested positive for cotinine using saliva samples while 74/380 (19%), 31/380 (9.0%), and 275/380 (72%) self-reported never, currently using, and to have ever used shisha, respectively.  Figure 2 shows the proportion who tested positive for cotinine across the year of study stratified by the university. Among the 74 participants who reported never to have used shisha, 14/74 (19%) tested positive for cotinine, 21/31 (68%) of those reported to be currently using shisha, and 77/275 (28%) of those reported to ever have used shisha tested positive for cotinine, respectively (Table 1). Among the 126 participants who self-reported use of other drugs apart from shisha, only 1/126 (0.8%) reported use of other tobacco products. Using the saliva test for cotinine as the gold standard, among those who reported to be currently using shisha, the sensitivity was 67.7% (95% CI: 48.6–83.3) and specificity was 73.9% (95% CI: 69.0–78.5) while among those who reported to ever have used shisha, the sensitivity was 87.5% (95% CI: 79.9–93.0) and specificity was 22.3% (95% CI: 17.5–27.9).

Among the 380 participants, 278 (73%) were male and the median (IQR) age was 22 (20 to 24) years. A total of 340/380 (89%) were single. Christianity (*n* = 237, 62%) was the most common religion while the majority (*n* = 242, 64%) were studying for a bachelor's degree. Approximately one-half (*n* = 193, 51%) were second-year students. More than one-third (*n* = 132, 35%) were enrolled in business-related courses. Very few (*n* = 24, 6.3%) had a monthly stipend >US$1890 with the majority having 119 (31%) < US$630 and 160 (42%) US$630 to 1260 (Table 2 which summarizes sociodemographic characteristics stratified by saliva test results for cotinine).

Regarding awareness levels on shisha, at least two-thirds of the respondents (*n* = 249, 66%) knew what shisha is but only 181 (48%) knew its contents. A total of 225 (59%) correctly mentioned the effects of shisha. Among the 306 who were currently or had ever used shisha, 25/306 (8.2%) were using shisha daily, 82/306 (27%) weekly, 106/306 (35%) monthly, and 93/306 (30%) regularly. Shisha was mostly used at the beginning of semester (*n* = 121/306, 40%). The most frequently reported effects experienced after using shisha were sexual urge (*n* = 76/306, 25%), anxiety (*n* = 38/306, 12%), coughing (*n* = 34/306, 11%), and hallucination (*n* = 33/306, 11%). Only 31/360 (10%) of those who reported use of shisha wished to be counselled to stop. More than half of those reported shisha use because of peer pressure (*n* = 167/306, 55%) while 44/306 (14%) because shisha was readily available and 30/306 (9.8%) to be awake (Table 3).

In the univariate analysis, being separated (crude risk ratio (CRR): 2.24 (95% CI: 1.54–3.24)) compared to being single was associated with testing cotinine positive. Religion (others vs. Islam (CRR: 0.80 (95% CI: 0.76–0.84))) was associated with testing cotinine positive. Compared to students studying diploma, studying for a degree (CRR: 1.53 (95% CI: 1.32–1.78)) was associated with cotinine positive. Compared to students in 1^st^ year, 2^nd^ years (CRR: 0.73 (95% CI: 0.56–0.95)) and 4^th^ years (CRR: 1.96 (95% CI: 1.42–2.70)) were associated with being cotinine positive. Compared to semester one of study, semester two (CRR: 0.75 (95% CI: 0.59–0.94)) was associated with testing cotinine positive. A monthly stipend of US$1260 to 1890 (CRR: 1.49 (95% CI: 1.14–1.96)) compared to <US$630 was associated with being cotinine positive. Knowledge of shisha (do you know what shisha is? CRR: 1.83 (95% CI: 1.17–2.86) and do you know what it contains? CRR: 1.60 (95% CI: 1.08–2.37)) was associated with cotinine positive. No other independent variable explored was associated with cotinine positive in the univariate analysis (Table 4).

In the multivariable analysis, being separated (adjusted risk ratio (aRR): 2.06 (95% CI: 1.45–2.94)) compared to being single was associated with testing cotinine positive. Compared to students studying diploma, studying for a degree (aRR: 1.32 (95% CI: 1.10–1.58)) was associated with cotinine positive. Compared to students in 1^st^ year, 3^rd^ years (aRR: 0.82 (95% CI: 0.68–0.98)) and 4^th^ years (aRR: 1.68 (95% CI: 1.22–2.33)) were associated with being cotinine positive. Knowledge of shisha (do you know what is shisha? aRR: 1.84 (95% CI: 1.18–2.87)) was associated with testing cotinine positive (Table 4).

Overall, 306/380 (81%, 95% CI: 76 to 84%) reported to either currently using or having ever used shisha and were considered as shisha users in regression analysis. In the univariate analysis, being married (crude risk ratio (CRR): 1.21 (95% CI: 1.03–1.42)) compared to being single was associated with shisha use. Religion (others vs. Islam (CRR: 1.11 (95% CI: 1.08–1.14))) was associated with shisha use. Compared to students in 4^th^ year or above, 2^nd^ years (CRR: 0.85 (95% CI: 0.78–0.93)) and 3^rd^ years (CRR: 0.79 (95% CI: 0.65–0.96)) were negatively associated with shisha use. No other independent variable explored was associated with shisha use in the univariate analysis (Table 5).

In the multivariable regression model, being married (adjusted risk ratio (aRR): 1.29 (95% CI: 1.07–1.57)) compared to being single was associated with shisha use. Religion (others vs. Islam (aRR: 1.07 (95% CI: 1.04–1.11))) was associated with shisha use. Compared to students in 4^th^ year or above, 1^st^ years (aRR: 1.08 (95% CI: 1.03–1.13)), 2^nd^ years (aRR: 0.88 (95% CI: 0.80–0.96)), and 3^rd^ years (CRR: 0.86 (95% CI: 0.78–0.95)) were associated with shisha use. Compared to students taking business courses, taking engineering courses (aRR: 1.21 (95% CI: 0.99–1.47)) had borderline effect. No other independent variable explored was associated with shisha use in the multivariable analysis (Table 5).

## 4. Discussion

This study reports 29% current shisha use based on testing positive for cotinine. The sensitivity of those who self-reported currently using shisha was about 68%, meaning more than two-thirds of them tested positive for cotinine while approximately 74% of those self-reported not to currently using shisha tested negative (specificity). This highlights the importance of saliva testing because relying on self-reporting alone would have misclassified 26% students as not using shisha, yet traces of cotinine were detected in their saliva.

Globally, assessment of variation between self-reported and performance of saliva testing with sensitivity and specificity values favours saliva testing as a more accurate tool for reporting tobacco use. For example, in Poland, about 5% of the participants who had been misclassified as nonsmokers based on self-reported current tobacco use were classified as smokers based on the cotinine cutoff value [31]. In India, self-reports among youths aged between 10 and 19 year had a low sensitivity (36.3%) and a positive predictive value of 72.6% [32]. It is therefore important to validate self-reports on tobacco use using biochemical markers presents in body fluids like saliva.

These results on current use of cotinine deviate from findings of a previous in the same region and similar subpopulation whereby 59% tested positive for cotinine [33]. Such a deviation was also observed in the study in Nairobi, Kenya, whereby 21.5% of the participants self-reported current shisha use [21]. A positive cotinine test among those who self-reported to have never used shisha could be due to poor memory recall, social desirability, passive smoking, or third-hand exposure. Both passive smoking also known as second-hand smoking and third-hand exposure are detrimental to human health [34, 35].

In this study, male students were more likely to be currently using shisha than their female counterparts; however, the difference was not statistically significant. These findings deviate from the results of a study at Jazan University where the prevalence rate of WTS was 34.0% and significantly higher in males (42.5%) than females at 27.0% [36]. The peak age group for shisha use was between 22 and 24 years. This is a deviation from a peak age of 25–29 in Rwanda [20], 21–25 in South Africa [22], and ages of initiation of 18–21 years among students in Jordan [37]. Participants of Christian faith were more likely to consume shisha. Such a high prevalence has previously been reported among students in two Kenyan universities [21, 33]. However, the number of Muslim students enrolled in both studies was extremely low and this could have caused a bias.

Based on current shisha use, more participants in the third year of study were more likely to use shisha by virtue of testing positive for cotinine. Such an incremental prevalence that is proportional to year of study has been reported among medical students in Istanbul, Türkiye, and in a South African medical school [38, 39]. Initiation is more likely to take place in first and second years of study by senior students who are already habitual users. Peer pressure, need to demonstrate adulthood, and academic stress could be the main drivers of shisha use in third year. Nonetheless, irrespective of the year of study, low cotinine test positivity rates were observed among participants from Moi University (Figure 2). This could be due to low number of participants or absence of environmental cues that trigger shisha use as discussed in the next paragraph. Participants from business-related studies were more likely to report current of shisha. Similar findings were reported in Rwanda [20] and Bangladesh [40]. Students enrolled in business-related studies are likely to use shisha due to idleness as the courses offered are relatively lighter.

Participants with a moderate monthly stipend of US$1260 to 1890 were more likely to use shisha. Similar findings have been reported in Taibah University in the KSA and Ankara in Türkiye [41, 42]. In general, students with higher socioeconomic status and having higher stipends per week had increased odds for shisha usage unlike other drugs where poverty is a risk factor. Participants from TUM were more likely to use shisha than those from KU and Moi universities. This could be due to the overrepresentation of participants enrolled from TUM or the presence of environmental cues within TUM. Environmental cues associated with recreational drugs may trigger cravings or a relapse, thus influencing the risk of shisha use [43].

Regarding awareness level, two-thirds of the participants knew the effects of shisha including those currently using shisha. These findings deviate from most studies in the MENA region where most consumers lacked knowledge on the effects of shisha [7, 11]. Despite knowing the negative health effects of nicotine, the participants were not deterred from consuming shisha. This could be due to the fact that most shisha manufacturers provide deceptive information [44]. Consequently there is need to upscale health education on the negative effects of shisha use in the wake of misinformation about commercial tobacco products. Most shisha consumers were most likely to consume shisha at the beginning of the semester partly due to free time or idleness. In Malaysia, a study reported shisha consumption among university students as a favourite pastime activity [45].

Findings on association between being married mirror the results of a study in the Qassim Region of KSA [18]. This could be due to marital stress or financial demands that come with marriage and hence the use of shisha to relax. Ironically, being separated was also a predictor of shisha use. This could be attributed to life post-separation which might be characterised by loneliness. Loneliness is a predictor of waterpipe use [46]. Despite having fewer Muslim participants, being a Muslim predicted shisha use. This can be attributed to Arabic culture since shisha is trendier in MENA and Eastern Mediterranean regions [5, 46, 47]. Relationship between being in year one to three of study and shisha use could be as a result of tobacco being a gateway drug [48].

The strength of this study is anchored on confirmed shisha consumption using oral fluids which is more reliable than self-reported shisha use. The findings of this study have shed more light on status of shisha use after the 2017 ban. The limitation of this study is the cross-sectional design which cannot establish evidence for temporal relationship between the exposures and shisha use. Additionally, use of the snowball sampling method could have introduced a selection bias. Future studies to consider using respondent driven sampling; use of hair samples and environmental toxicology to detect shisha use up to a window period of six months retrospectively while the later can detect third-hand exposure.

## 5. Conclusion

Nearly one-third of university students along the Kenyan coast are active shisha users. Saliva testing for cotinine is a more reliable method of reporting tobacco use. We recommend upscaling of health education, re-enforcement of the current ban on shisha consumption by concerned authorities, and saliva testing for cotinine while assessing current tobacco use.

## Figures and Tables

**Table 1:** Self-reported and confirmed shisha use among students in public universities in Coastal Kenya in 2018-2019.

Exposure (*N* = 380)	Shisha use	
	Self-reported use, *n* = 306 (81%)	Cotinine positive, *n* = 112 (29%)
Ever used	275 (72%)	77 (28%)^∗^
Current use	31 (9.0%)	21 (68%)^∗^
Never used	74 (19%)	14 (19%)^∗^

^∗^Proportions of participants who self-reported shisha usage and tested positive for cotinine. For example for ever used 77/275 (28%), 21/31 (68%) and 14/74 (19%) for current and never used respectively.

**Table 2:** Sociodemographic characteristics and cotinine test results based on saliva analysis among students in public universities in Coastal Kenya between 2018 and 2019.

	All students (*N* = 380)	Cotinine test status		*P* value^∗^
		Negative (*n* = 268)	Positive (*n* = 112)	
*Sex*				
Male	278 (73)	185 (69)	93 (83)	0.005
Female	102 (27)	83 (31)	19 (17)	

*Age in years*				
18 to 21	129 (34)	90 (33)	39 (35)	0.71
22 to 24	154 (41)	112 (42)	42 (37)	
≥25	97 (26)	66 (25)	31 (28)	

*Marital status*				
Single	340 (89)	245 (91)	95 (85)	0.01
Married	24 (6.3)	17 (6.3)	7 (6.3)	
Separated	16 (4.2)	6 (2.2)	10 (8.9)	

*Religion*				
Islam	132 (35)	87 (32)	45 (40)	0.35
Christianity	237 (62)	173 (65)	64 (57)	
Others	11 (2.9)	8 (3.0)	3 (2.7)	

*Education level*				
Diploma	119 (31)	93 (35)	26 (23)	0.07
Degree	242 (64)	161 (60)	81 (72)	
Postgraduate	19 (5.0)	14 (5.2)	5 (4.5)	

*Year of study*				
1^st^	64 (17)	44 (16)	20 (18)	<0.001
2^nd^	193 (51)	149 (56)	44 (39)	
3^rd^	87 (23)	61 (23)	26 (23)	
4^th^ and above	36 (9.5)	14 (5.2)	22 (20)	

*Semester of the year*				
Semester 1	200 (53)	133 (50)	67 (60)	0.07
Semester 2	180 (47)	135 (50)	45 (40)	

*Course enrolled*				
Business	132 (35)	102 (38)	30 (27)	0.34
Engineering	52 (14)	35 (13)	17 (15)	
Applied sciences	56 (15)	38 (14)	18 (16)	
Computer science	81 (21)	54 (20)	27 (24)	
Social sciences	59 (16)	39 (15)	20 (18)	

*Pocket money per month (US$)*				
<630	119 (31)	89 (33)	30 (27)	0.12
630 to 1260	160 (42)	117 (44)	43 (38)	
1260 to 1890	77 (20)	48 (20)	29 (26)	
>1890	24 (6.3)	14 (5.2)	10 (8.9)	

*Recruiting university*				
TUM	220 (58)	142 (53)	78 (70)	0.003
MOI	60 (16)	52 (19)	8 (7.1)	
KU	100 (26)	74 (28)	26 (23)	

^∗^ *P* values from chi-square/Fisher's exact test; all results are expressed as frequency and proportion; US dollar exchange rate was KES 125.9353 per one US dollar in January to February 2023 (https://www.centralbank.go.ke/rates/forex-exchange-rates/).

**Table 3:** Awareness levels on shisha use by students in public universities in Coastal Kenya between 2018 and 2019.

	All students (*N* = 380)	Shisha consumption			*P* value^∗^
		Never use (*n* = 74)	Current use (*n* = 31)	Ever use (*n* = 275)	
*Do you know what shisha is?*					
No	131 (34)	22 (16.8)	8 (6.1)	101 (77.1)	0.30
Yes	249 (66)	52 (20.9)	23 (9.2)	174 (69.9)	

*Do you know what it contains?*					
No	114 (30)	24 (21.1)	9 (7.9)	81 (71.1)	0.45
Yes	181 (48)	30 (16.6)	18 (9.9)	133 (73.5)	
Not sure	85 (22)	20 (23.5)	4 (4.7)	61 (71.8)	

*Do you know the effects of shisha?*					
No	155 (41)	44 (28.4)	8 (5.2)	103 (66.5)	**0.001**
Yes	225 (59)	30 (13.3)	23 (10.2)	172 (76.4)	

*Frequency of using shisha*					
Daily	25 (8.2)	—	2 (8.0)	23 (92.0)	0.14
Weekly	82 (27)	—	7 (8.5)	75 (91.5)	
Monthly	106 (35)	—	7 (6.6)	99 (93.4)	
Regularly	93 (30)	—	15 (16.1)	78 (83.9)	

*Time used in the semester*					
Beginning of semester	121 (40)	—	4 (3.3)	117 (96.7)	**<0.001**
Mid of semester	52 (17)	—	1 (1.9)	51 (98.1)	
End of semester	78 (25)	—	7 (9.0)	71 (91)	
Throughout the semester	55 (18)	—	19 (34.6)	36 (65.6)	

*What effects do you experience after using shisha?*					
Anxiety	38 (12)	—	6 (15.8)	32 (84.2)	0.40
Coughing	34 (11)	—	3 (8.8)	31 (91.2)	
Hallucination	33 (11)	—	1 (3.0)	32 (96.9)	
High appetite	30 (9.8)	—	4 (13.3)	26 (86.7)	
No appetite	19 (6.2)	—	3 (15.8)	16 (84.2)	
Tired	17 (5.6)	—	0	17 (100)	
Sexual urge	76 (25)	—	10 (13.2)	66 (86.8)	
Emotional	29 (9.5)	—	1 (3.5)	28 (96.6)	
Don't know	30 (9.8)	—	3 (10.0)	27 (90.0)	

*Would you like to be counselled to stop taking shisha?*					
No	275 (90)	—	28 (10.2)	247 (89.8)	0.93
Yes	31 (10)	—	3 (9.7)	28 (90.3)	

*Where do you get shisha from?*					
Home	32 (10)	—	5 (15.6)	27 (84.4)	0.32
Tudor	29 (9.5)	—	0	29 (100)	
Kongowea	13 (4.3)	—	1 (7.7)	12 (92.3)	
Mtwapa	63 (21)	—	6 (9.5)	57 (90.5)	
Others	169 (55)	—	19 (11.2)	150 (88.8)	

*Reasons for using shisha*					
Peer pressure	167 (55)	—	14 (8.4)	153 (91.6)	0.29
Family background	24 (7.8)	—	5 (20.8)	19 (79.2)	
Availability	44 (14)	—	8 (18.2)	36 (81.8)	
Money availability	8 (2.6)	—	1 (12.5)	7 (87.5)	
Failure at school	11 (3.6)	—	1 (9.1)	10 (90.9)	
Stress	9 (2.9)	—	0	9 (100)	
Mass media influence	10 (3.3)	—	1 (10.0)	9 (90.0)	
To be awake	30 (9.8)	—	1 (3.3)	29 (96.7)	
Others	3 (1.0)	—	0	3 (100)	

*Do you use other drugs?*					
No	254 (67)	54 (21.3)	15 (5.9)	185 (72.8)	**0.05**
Yes	126 (33)	20 (15.9)	16 (12.7)	90 (71.4)	

^∗^ *P* values from chi-square/Fisher's exact test; all results are expressed as frequency and proportion; 74 participants who never reported use of shisha were excluded in the analysis. Statistical significance at *α* < 0.05.

**Table 4:** Univariate and multivariable analysis of factors associated with cotinine positive among students in public universities in Coastal Kenya between 2018 and 2019.

	Cotinine positive (*N* = 112)	Univariate analysis		Multivariable analysis	
		Crude risk ratio (CRR)	*P* value	Adjusted risk ratio (aRR)	*P* value
*Sex*					
Male	93 (83)	Reference		¶	
Female	19 (17)	0.56 (0.17–1.85)	0.34	¶	

*Age in years*					
18 to 21	39 (35)	Reference		¶	
22 to 24	42 (38)	0.90 (0.57–1.44)	0.67	¶	
≥25	31 (28)	1.06 (0.58–1.92)	0.86	¶	

*Marital status*					
Single	95 (85)	Reference		Reference	
Married	7 (6.3)	1.04 (0.37–2.93)	0.94	1.03 (0.41–2.57)	0.95
Separated	10 (8.9)	2.24 (1.54–3.24)	**<0.001**	2.06 (1.45–2.94)	**<0.001**

*Religion*					
Islam	45 (40)	Reference		¶	
Christianity	64 (57)	0.79 (0.47–1.33)	0.38	¶	
Others	3 (2.7)	0.80 (0.76–0.84)	**<0.001**	¶	

*Education level*					
Diploma	26 (23)	Reference		Reference	
Degree	81 (72)	1.53 (1.32–1.78)	**<0.001**	1.32 (1.10–1.58)	**0.003**
Postgraduate	5 (4.5)	1.20 (0.65–2.23)	0.55	1.17 (0.63–2.18)	0.63

*Year of study*					
1^st^	20 (18)	Reference		Reference	
2^nd^	44 (39)	0.73 (0.56–0.95)	0.02	0.68 (0.45–1.04)	0.08
3^rd^	26 (23)	0.96 (0.69–1.33)	0.79	0.82 (0.68–0.98)	0.03
4^th^ and above	22 (20)	1.96 (1.42–2.70)	**<0.001**	1.68 (1.22–2.33)	**0.002**

*Semester of the year*					
Semester 1	67 (60)	Reference		¶	
Semester 2	45 (40)	0.75 (0.59–0.94)	0.01	¶	

*Course enrolled*					
Business studies	30 (27)	Reference		¶	
Engineering	17 (15)	1.44 (0.99–2.09)	0.06	¶	
Applied and health sciences	18 (16)	1.41 (0.82–2.44)	0.21	¶	
Computer sciences	27 (24)	1.47 (0.47–4.56)	0.51	¶	
Social sciences	20 (18)	1.49 (0.53–4.23)	0.45	¶	

*Stipend per month (US$)*					
<630	30 (27)	Reference		¶	
630 to 1260	43 (38)	1.07 (0.79–1.44)	0.68	¶	
1260 to 1890	29 (26)	1.49 (1.14–1.96)	0.004	¶	
>1890	10 (8.9)	1.65 (0.85–3.21)	0.14	¶	

*Do you know what is shisha?*					
No	25 (22)	Reference		Reference	
Yes	87 (78)	1.83 (1.17–2.86)	**0.008**	1.84 (1.18–2.87)	**0.007**

*Do you know what it contains?*					
No	66 (59)	Reference		¶	
Yes	26 (23)	1.60 (1.08–2.37)	0.02	¶	
Not sure	20 (18)	1.03 (0.60–1.77)	0.91	¶	

*Do you know the effects of shisha?*					
No	36 (32)	Reference		¶	
Yes	76 (68)	1.45 (0.84–2.51)	0.18	¶	

¶;variables not selected for inclusion in the multivariable regression model, risk ratios from a log-binomial regression models, US dollars exchange rate was KES 125.9353 per one US dollar in January to February 2023 (https://www.centralbank.go.ke/rates/forex-exchange-rates/). Statistical significance at *α* < 0.05.

**Table 5:** Univariate and multivariable analysis of factors associated with reported shisha use by students in public universities in Coastal Kenya between 2018 and 2019.

	Shisha use (*N* = 306)	Univariate analysis		Multivariable analysis	
		Crude risk ratio (CRR)	*P* value	Adjusted risk ratio (aRR)	*P* value
*Sex*					
Male	228 (75)	Reference		¶	
Female	78 (25)	0.93 (0.68–1.29)	0.67	¶	

*Age in years*					
18 to 21	117 (38)	Reference		¶	
22 to 24	122 (40)	0.87 (0.63–1.21)	0.41	¶	
≥25	67 (22)	0.76 (0.53–1.10)	0.15	¶	

*Marital status*					
Single	269 (88)	Reference		Reference	
Married	23 (7.5)	1.21 (1.03–1.42)	0.02	1.29 (1.07–1.57)	0.009
Separated	14 (4.6)	1.11 (0.86–1.42)	0.43	1.04 (0.84–1.30)	0.70

*Religion*					
Islam	119 (39)	Reference		Reference	
Christianity	176 (58)	0.82 (0.63–1.07)	0.15	0.82 (0.65–1.02)	0.07
Others	11 (3.6)	1.11 (1.08–1.14)	<0.001	1.07 (1.04–1.11)	<0.001

*Education level*					
Diploma	94 (31)	Reference		¶	
Degree	199 (65)	1.04 (0.95–1.14)	0.39	¶	
Postgraduate	13 (4.3)	0.87 (0.61–1.23)	0.42	¶	

*Year of study*					
1^st^	60 (20)	1.02 (0.98–1.07)	0.29	1.08 (1.03–1.13)	0.002
2^nd^	150 (49)	0.85 (0.78–0.93)	<0.001	0.88 (0.80–0.96)	0.003
3^rd^	63 (21)	0.79 (0.65–0.96)	0.02	0.86 (0.78–0.95)	0.004
4^th^ and above	33 (11)	Reference		Reference	

*Semester of the year*					
Semester 1	160 (52)	Reference		¶	
Semester 2	146 (48)	1.01 (0.82–1.26)	0.90	¶	

*Course enrolled*					
Business studies	98 (32)	Reference		Reference	
Engineering	50 (16)	1.30 (0.99–1.70)	0.06	1.21 (0.99–1.47)	0.05
Applied and health sciences	49 (16)	1.18 (0.91–1.53)	0.21	1.09 (0.89–1.34)	0.40
Computer sciences	62 (20)	1.03 (0.87–1.21)	0.72	1.05 (0.89–1.25)	0.58
Social sciences	47 (15)	1.07 (0.87–1.32)	0.50	1.01 (0.85–1.19)	0.95

*Stipend per month (US$)*					
<630	100 (33)	Reference		¶	
630 to 1260	132 (43)	0.98 (0.88–1.09)	0.74	¶	
1260 to 1890	57 (19)	0.88 (0.69–1.12)	0.30	¶	
>1890	17 (5.6)	0.84 (0.53–1.35)	0.48	¶	

*Do you know what shisha is?*					
No	109 (36)	Reference		¶	
Yes	197 (64)	0.95 (0.83–1.09)	0.49	¶	

*Do you know what it contains?*					
No	151 (49)	Reference		¶	
Yes	90 (29)	0.95 (0.72–1.24)	0.69	¶	
Not sure	65 (21)	0.92 (0.67–1.24)	0.57	¶	

*Do you know the effects of shisha?*					
No	111 (36)	Reference		¶	
Yes	195 (64)	1.21 (0.89–1.64)	0.22	¶	

¶;variables not selected for inclusion in the multivariable regression model, risk ratios from a log-binomial regression models, US dollars exchange rate was KES 125.9353 per one US dollar in January to February 2023 (https://www.centralbank.go.ke/rates/forex-exchange-rates/).

## Data Availability

The datasets analyzed during the current study are available from the corresponding author on reasonable request.

## Abbreviations

Sudan: Refers to Republic of the Sudan and the Republic of South Sudan
South Africa: Republic of South Africa
Cotinine: Is an alkaloid found in tobacco and is also the predominant metabolite of nicotine
Semester: A fixed period of time in the academic year in the university during which lectures, seminars, practicals, and other courses take place (in our setup, a semester lasts for 14 weeks)
Diploma: Diploma programmes are designed to provide hands-on training for a particular trade or profession (in our setup, a diploma programme runs for 2 to 3 years depending on the course)
Degree: An undergraduate bachelor's degree is a higher academic award than a diploma (in the context of our study, degree programmes typically take four or seven years of full-time study to complete depending on the course)
Postgraduate: A postgraduate degree includes a range of qualifications completed after a bachelor's degree (in the context of this study, this includes courses at Masters and Doctorate levels).
